# High-resolution crystal structures of a myxobacterial phytochrome at cryo and room temperatures

**DOI:** 10.1063/1.5120527

**Published:** 2019-09-17

**Authors:** Juan C. Sanchez, Melissa Carrillo, Suraj Pandey, Moraima Noda, Luis Aldama, Denisse Feliz, Elin Claesson, Weixiao Yuan Wahlgren, Gregory Tracy, Phu Duong, Angela C. Nugent, Andrew Field, Vukica Šrajer, Christopher Kupitz, So Iwata, Eriko Nango, Rie Tanaka, Tomoyuki Tanaka, Luo Fangjia, Kensuke Tono, Shigeki Owada, Sebastian Westenhoff, Marius Schmidt, Emina A. Stojković

**Affiliations:** 1 Department of Biology, Northeastern Illinois University, 5500 N. St. Louis Ave., Chicago, Illinois 60625, USA; 2 Physics Department, University of Wisconsin-Milwaukee, 3135 N. Maryland Ave., Milwaukee, Wisconsin 53211, USA; 3 Department of Chemistry and Molecular Biology, University of Gothenburg, Box 462, 40530 Gothenburg, Sweden; 4 The University of Chicago, Center for Advanced Radiation Sources, 9700 South Cass Ave., Bldg 434B, Argonne, Illinois 60439, USA; 5 Department of Cell Biology, Graduate School of Medicine, Kyoto University, Yoshidakonoe-cho, Sakyo-ku, Kyoto 606-8501, Japan; 6 RIKEN SPring-8 Center, 1-1-1, Kouto, Sayo-cho, Sayo-gun, Hyogo 679-5148, Japan; 7 Japan Synchrotron Radiation Research Institute, 1-1-1 Kouto, Sayo-cho, Sayo-gun, Hyogo 679-5198, Japan

## Abstract

Phytochromes (PHYs) are photoreceptor proteins first discovered in plants, where they control a variety of photomorphogenesis events. PHYs as photochromic proteins can reversibly switch between two distinct states: a red light (Pr) and a far-red light (Pfr) absorbing form. The discovery of Bacteriophytochromes (BphPs) in nonphotosynthetic bacteria has opened new frontiers in our understanding of the mechanisms by which these natural photoswitches can control single cell development, although the role of BphPs *in vivo* remains largely unknown. BphPs are dimeric proteins that consist of a photosensory core module (PCM) and an enzymatic domain, often a histidine kinase. The PCM is composed of three domains (PAS, GAF, and PHY). It holds a covalently bound open-chain tetrapyrrole (biliverdin, BV) chromophore. Upon absorption of light, the double bond between BV rings C and D isomerizes and reversibly switches the protein between Pr and Pfr states. We report crystal structures of the wild-type and mutant (His275Thr) forms of the canonical BphP from the nonphotosynthetic myxobacterium *Stigmatella aurantiaca* (*Sa*BphP2) in the Pr state. Structures were determined at 1.65 Å and 2.2 Å (respectively), the highest resolution of any PCM construct to date. We also report the room temperature wild-type structure of the same protein determined at 2.1 Å at the SPring-8 Angstrom Compact free electron LAser (SACLA), Japan. Our results not only highlight and confirm important amino acids near the chromophore that play a role in Pr-Pfr photoconversion but also describe the signal transduction into the PHY domain which moves across tens of angstroms after the light stimulus.

## INTRODUCTION

**FIG. 1.**
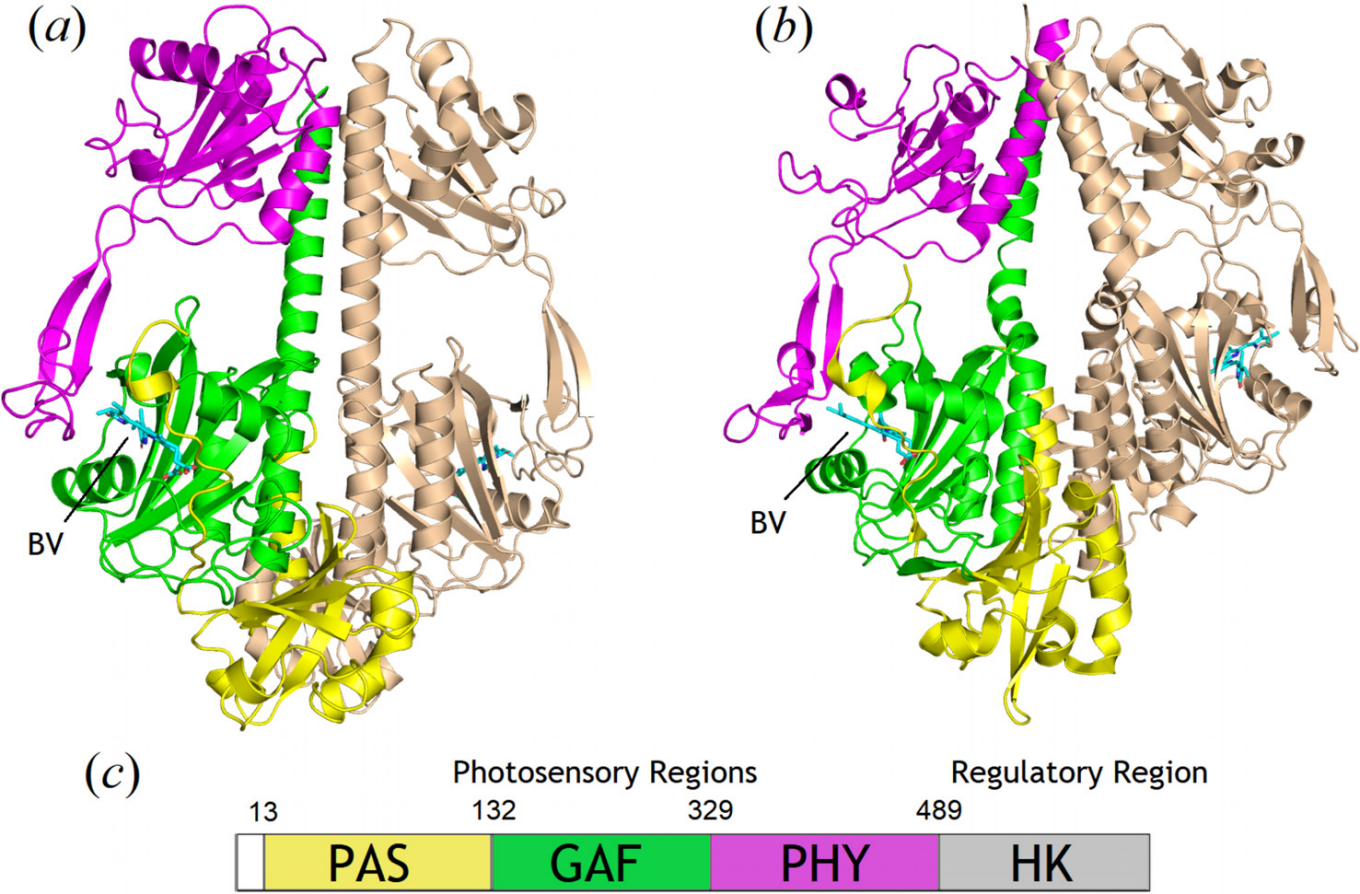
Comparisons of the *Sa*BphP2 PCM (a) in the wild-type and *Sa*BphP1 PCM (b) in the wild-type forms. The PAS, GAF, and PHY domains are colored yellow, green, and magenta, respectively. The PCM is a dimer with one monomer highlighted in gold. The kink at the helical transition from GAF to PHY is apparent in panel (b) and BV is marked in panels (a) and (b). (c) Schematic presentation of domain organization of BphPs is below with sequence numbers provided for *Sa*BphP2.

Phytochromes (PHYs) are red-light sensing enzymatic switches first discovered in plants, with homologs in the photosynthetic and nonphotosynthetic bacteria. PHYs are composed of a photosensory core module (PCM) and an effector, enzymatic domain. In bacteriophytochromes (BphPs), the PCM consists of three domains named PAS (Per ARNT Sim), GAF (cGMP phosphodiesterase/adenylyl cyclase/FhIA), and PHY (phytochrome-specific GAF-related) [Fig. 1(a)].^1–5^ The effector domain is typically a histidine kinase, covalently linked to a PHY domain, although other nonenzymatic output domains can be found.^6,7^ Together, the PCM and effector domains are able to elicit an array of important physiological responses upon absorption of red light, due to a covalently bound-bilin based chromophore [Fig. 1(b)]. In photosynthetic bacteria, such as *Rhodopseudomonas palustris*, BphPs regulate the synthesis of light-harvesting complexes, which are essential in photosynthesis.^8,9^ However, the role of BphPs in nonphotosynthetic bacteria is less understood. Some nonphotosynthetic bacteria, such as *Deinococcus radiodurans*, use BphPs to regulate the production of carotenoid pigments, a vital defense mechanism against harmful light exposure.^10^ Others are involved in conjugation,^11^ plant colonization,^12^ and quorum sensing.^13^ We recently determined the crystal structures of an unusual BphP from nonphotosynthetic myxobacterium *Stigmatella aurantiaca,* denoted *Sa*BphP1, which lacks a conserved histidine in the chromophore binding pocket and identified its important functional role in the unique photomorphogenic response of this micro-organism.^5,14^ Myxobacteria are distinguished among prokaryotes by a multicellular stage in their life cycle known as fruiting bodies, which in *S. aurantiaca* is controlled by light.^15–17^

**FIG. 2.**
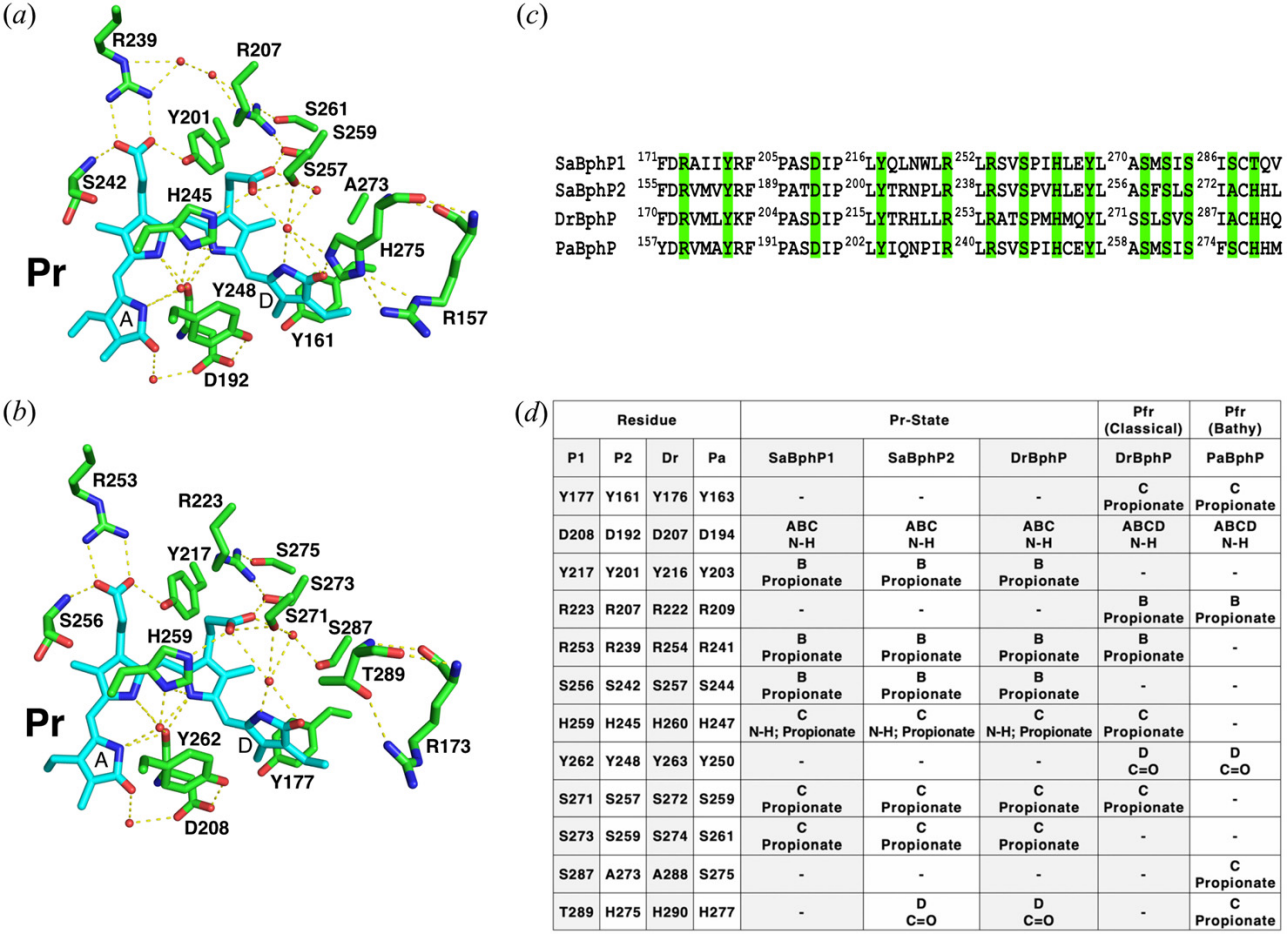
Hydrogen-bonding network of the conserved amino acids and neighboring water molecules stabilizing BV chromophore in the Pr state of *Sa*BphP2 wild-type (a) and *Sa*BphP1 wild-type (b). Hydrogen bonds are marked with dashed lines, and amino acids located in the GAF domain are highlighted in green. (c) Partial protein sequence alignment highlighting conserved amino acids stabilizing propionate side chains of BV chromophore (in cyan) as well as A-D pyrrole rings. (d) Table with conserved amino acids from *Sa*BphP1, *Sa*BphP2, *Dr*BphP, and bathy *Pa*BphP with specific BV interactions as identified in the Pr and Pfr states.

Interestingly, *S. aurantiaca* also contains a second, canonical phytochrome (*Sa*BphP2) which has a different photochemistry from *Sa*BphP1. However, both proteins bind the same bilin chromophore and share a large amino acid sequence identity (38%).^5,14,18^ Biliverdin (BV) is an open-chain tetrapyrrole that is highly conjugated and allows for reversible photoconversion in BphPs between the red light absorbing (Pr, λ_max_ 700 nm) and far-red light absorbing (Pfr, λ_max_ 750 nm) states.^19^ The ability to form and photoconvert reversibly between two stable absorbing states allows phytochromes to act as light-regulated switches in many physiological processes, a characteristic that is unique among photoreceptors. BV is covalently bound to the PAS domain via a thioether linkage to a conserved cysteine and is characterized by a series of hydrogen bonds between its four pyrrole rings and amino acids within the GAF domain.^2,20^ Photoexcitation of BV leads to the Z/E isomerization of the C15=C16 double bond located between the C and D rings [Fig. 2(b)].^21,22^ As a result, a rotation of the D-ring leads to the formation of new hydrogen bonds around the chromophore, which causes major conformational changes throughout the polypeptide chains. These changes result in two distinct structures, one in the dark-adapted Pr state for the classical phytochrome which upon red-light exposure switches into Pfr and the other in the dark-adapted Pfr state for the bathy phytochromes which upon far-red light exposure reverts to Pr.^23–26^

Key to the phytochrome function is the primary photoresponse on picosecond and nanosecond timescales, which defines the moment at which light signals are translated into conformational changes ultimately impacting enzymatic activity.^27^ The conformational changes are currently not well understood due to the limited number of structures at the atomic resolution in the Pr and Pfr states and/or directly after photoexcitation of the same BphP.

Structural studies of BphPs are necessary in order to understand intricate details of BphP photochromism in real time, on ps to ms time scales. Furthermore, BphPs have been optimized as infrared fluorescent protein markers (IFPs) for *in vivo* imaging of internal organs in mammals.^28^ IFPs readily bind BV, abundant in mammalian cells due to heme metabolism, and are readily detectable through the skin, avoiding invasive surgical procedures. However, engineered, BphP-derived IFPs still have a fairly low fluorescence quantum yield (<14%)^29^ that could be improved with the better understanding of the initial steps in the BphP photocycle. Here, we report three crystal structures of the *Sa*BphP2 photosensory core module (PCM), lacking the effector histidine kinase domain. The reported structures are of a *Sa*BphP2 PCM in the wild-type (wt) and mutant forms determined at 1.65 Å and 2.2 Å, respectively. This is the highest resolution structure of any PCM construct in the wild-type form to date, revealing the important intricate details of the hydrogen-bonding network of the conserved amino acids and waters stabilizing BV chromophore in the Pr state. To assess the impact of both temperature and X-ray dose on the two structures of *Sa*BphP2 and complement our earlier studies on the related *Sa*BphP1 PCM, we also determined the ambient temperature structure of wild-type *Sa*BphP2 PCM, essentially free of radiation damage as a result of using an X-ray free electron laser (XFEL).

## MATERIALS AND METHODS

### Protein expression and purification

The coding region for residues 1–497 of the wild-type *Sa*BphP2 was amplified from *S. aurantiaca* DW4/3‐1 genomic DNA by polymerase chain reaction (PCR). The DNA was a gift from Dr. Rolf Müller (Saarland University, Saarbrücken, Germany), cut by restriction enzymes NdeI and HindIII (New England Biolabs, Beverly, MA), and ligated into the corresponding sites of the expression vector pET28c(+) (Invitrogen, Carlsbad, CA). The following pairs of primers were used to PCR-amplify the coding region of *Sa*BphP2 PCM. Forward: 5′-GATACATATGCCCCCGTCCGTCTCTGAACTC-3′ and reverse: 5′-GATCAAGCTTAGGCCTGGCG CAGCAC-3′.

*E. coli* containing the vector of *Sa*BphP2 PCM were coexpressed with heme oxygenase from plasmid pET11a in BL21 cells. Cells were grown aerobically at 37 °C to 3 × 10^8^ cells/ml and induced at 18 °C for 16–18 h using 1 mM isopropyl-β-d-thiogalactopyranoside and 0.5 mM δ-aminolevulinic acid. Cells were centrifuged at 4000 rpm for 10 min at 4 °C. Cells were resuspended in 150 mM NaCl, 20 mM Tris-HCl pH 8.0, and 15% v/v glycerol with protease inhibitor cocktail (Roche). Cells were lysed using sonication and centrifuged at 12 000 rpm for 1 h at 4 °C. The supernatant was transferred and protein was stabilized with exogenous 20 *μ*M BV in dimethylsulfoxide (DMSO) for 1 h at 4 °C. The supernatant solution was added to Talon Co^2+^ metal ion affinity chromatography columns (Takara Clontech). The protein was eluted with 300 mM imidazole, 20 mM NaCl, and 20 mM Tris-HCl pH 8.0. Eluted protein was further purified via Amicon filtration (10 kDa cutoff) at 4000 rpm for 20 min, 3 times. All steps were performed under green safety lights. The protein size and purity were analyzed via silver-stained 10% sodiumdodecylsulfate polyacrylamide gel electrophoresis (SDS-PAGE).

### UV-visible spectrum absorbance spectroscopy

Solution samples were illuminated using light interference filters of 660 nm and 750 nm, respectively, with a 10 nm bandwidth (ANDOVER, Salem, MA), and the spectra were assayed at room temperature from 240 to 800 nm with a Hitachi 3130 spectrophotometer (Hitachi, Tokyo, Japan). Absorption spectra of the crushed *Sa*BphP2 wild-type crystals were collected at room temperature using a microspectrophotometer at the BioCARS facility at the Advanced Photon Source. Crystal spectra were collected before and after illumination with 660 nm light from a high power light emitting diode (Thorlabs) for 1 min at ∼5 mW/mm^2^. The spectra were normalized and displayed, and difference spectra were calculated with the free plotting tool Xmgrace.

### Protein crystallization

Crystallization conditions were identified using cryoscreens from Hampton Research (HR2‐121 and HR2‐122). Crystallization conditions were further optimized via a 96-well additive screen (Hampton Research HR2‐428). Screens produced *Sa*BphP2 PCM crystals of 100 × 100 *μ*m^2^. Final crystallization conditions (mother liquor) consisted of both cryoscreen solution and additive. Mother liquor consisted of 425 *μ*l 0.17 M Ammonium acetate, 0.085 M Sodium citrate tribasic dihydrate pH 5.6, 25.5% w/v Polyethylene glycol 4000, 15% v/v Glycerol (cryoscreen solution), and 75 *μ*l 20% w/v Benzamidine Hydrochloride (as an additive). Crystals were obtained by the hanging drop vapor diffusion method in dark conditions at 16 °C with a protein concentration of 30 mg/ml. For the serial femtosecond crystallographic experiments, microcrystals of the *Sa*BphP2 PCM wild-type were prepared by mixing 60 mg/ml of protein with a 2:3 ratio of the same precipitant as listed above. The mixture was seeded with a 2 *μ*l slurry of crushed macrocrystals prepared using glass beads from JenaBioscience and added to the sitting drops. After 4 days of incubation, the microcrystals were collected and concentrated to about 10^11^ crystals/ml and subsequently folded into a tenfold amount of nuclear grade grease.^30,31^ All steps in crystallization and tray observations were performed under green safety light.

### X-ray crystallography and data collection

X-ray data were collected on macroscopic *Sa*BphP2-wt and H275T mutant PCM crystals at Sector 19, ID-D (Structural Biology Center) of the Advanced Photon Source at cryogenic temperatures (100 K) and processed by HKL3000.^32^ Data at room temperature were collected from the *Sa*BphP2 PCM-wt microcrystal–grease mixture at beamline BL2^33^ at the Spring-8 Angstrom Compact free electron LAser (SACLA, RIKEN SPring-8 Center, Japan) during a crystal screening beamtime (6 h) in April 2019. Up to 200 *μ*l of the mixture were transferred into an injector reservoir and extruded into air at ambient temperatures (293 K) through a 100 *μ*m wide nozzle with a flow rate of about 0.2 *μ*l/min. The stream of microcrystals was exposed to intense X-ray pulses of <10 fs duration with a 30 Hz repetition rate. Diffraction patterns were collected on a CCD detector with eight modules^34^ and analyzed with a user-friendly data-processing pipeline^35^ consisting of hit-finding with Cheetah^36^ and indexing and Monte Carlo integration by CrystFEL.^37^ The hit rate was about 35%. About 54% of diffraction patterns were successfully indexed. Both Mosflm and DirAx were used for indexing. The extracted partial intensities were merged to full reflection intensities using the “partialator” in CrystFEL. For data statistics, see supplementary material Table 1. The full intensities were converted to structure-factor amplitudes by software based on the CCP4 suite of programs.^38^

### Structure determination

The *Sa*BphP2 PCM crystalizes in space group P2_1_ with a homodimer in the asymmetric unit (supplementary material Table 1). Initially, the *Sa*BphP2 crystals were severely twinned, which compromised data quality and data interpretability. A molecular replacement solution could be found using the structure of the *Dr*BphP PCM, subunit A, as a search model. Molecular replacement was performed using “Phaser.”^39^ Two solutions for the search model were found which orient to each other head to head like in a typical BphP. The molecular replacement solution was used to phase data to higher resolution (2.2 Å). An initial model was determined using the “Autobuild” option in the program PHENIX.^40^ This model substantially deviated from the molecular replacement model and showed for the first time long straight helices along the dimer interface of the *Sa*BphP2 PCM. Subsequently, data to 1.9 Å and 2.2 Å for wild-type and the H275T mutant, respectively, were free of twinning due to the addition of benzamidine to the crystallization buffer. The initial model was completed by manually retracing some of the large loops in COOT^41^ and correcting the positions of the C-terminal helices. Data to 1.65 Å were finally collected on the SaBphP2-wt PCM. These data were used to refine a final model and determine the positions of more than 1100 water molecules. Our structure shows clear evidence for the sp3 hybridization of the ring A carbon number 2 after binding of the ring A vinyl to the Cys-13 sulfur (supplementary material Fig. 3, inset). The *Sa*BphP2-wt model was used to refine the structures of the His275Thr mutant. Clear negative difference electron density on His-275 indicated that the crystals were grown from His275Thr mutant protein. Histidine was replaced with Threonine in the structure and refined. The room temperature *Sa*BphP2-wt PCM structure was determined with the P2 model determined at 100 K as the initial model. After rigid body refinement and several rounds of restrained refinement in Phenix, R-factors settle below 20% and are acceptable (31%) even at 2.1 Å. Notably, in between the PHY domains of subunits A and B, strong positive electron density indicated the presence of a benzamidine molecule which was added to the crystallization buffer. Benzamidine cannot be identified in the cryotemperature structures and is most likely squeezed out between the PHY domains during the freezing process. The role of this additive in the crystal quality improvement now becomes clear. It intercalates between the PHY domains and stabilizes the structure and reduces structural heterogeneity. The structure of the benzamidine molecule was refined in a final step with that of the room temperature *Sa*BphP2 PCM. Structural illustrations were generated with the programs PyMol (https://pymol.org/2/) and UCSF Chimera.^42^

## RESULTS

### Crystal structures of SaBphP2 at cryo and room temperatures

To gain a detailed understanding of the function of the *S. aurantiaca* phytochromes, we examined the crystal structures of *Sa*BphP2 PCM at cryo and room temperatures. The wild-type protein crystallized in the dark in the Pr state in space group P2_1_. Crystals grown from the PCM constructs diffracted to 1.65 Å resolution at 100 K and 2.1 Å at 293 K (supplementary material Table 1). In the *Sa*BphP2 PCM crystals, subunits A and B form a noncrystallographic dimer. The structure of the *Sa*BphP2 PCM displays the typical PAS-GAF-PHY domain architectures highlighted in yellow, green, and magenta in Fig. 1, respectively. The BV chromophore is bound to Cys13 close to the N-terminus. The N-terminal amino-acid sequence threads through a loop composed of GAF domain amino acids and forms the knot characteristic of all phytochrome structures.^2,4^ At around residue 340, the GAF domain transitions to the PHY domain. At residue 325, the helical structure in the *Sa*BphP1 wild-type is kinked in both subunits [Fig. 1(b)]. This kink is absent in all *Sa*BphP2 structures determined here.

**FIG. 3.**
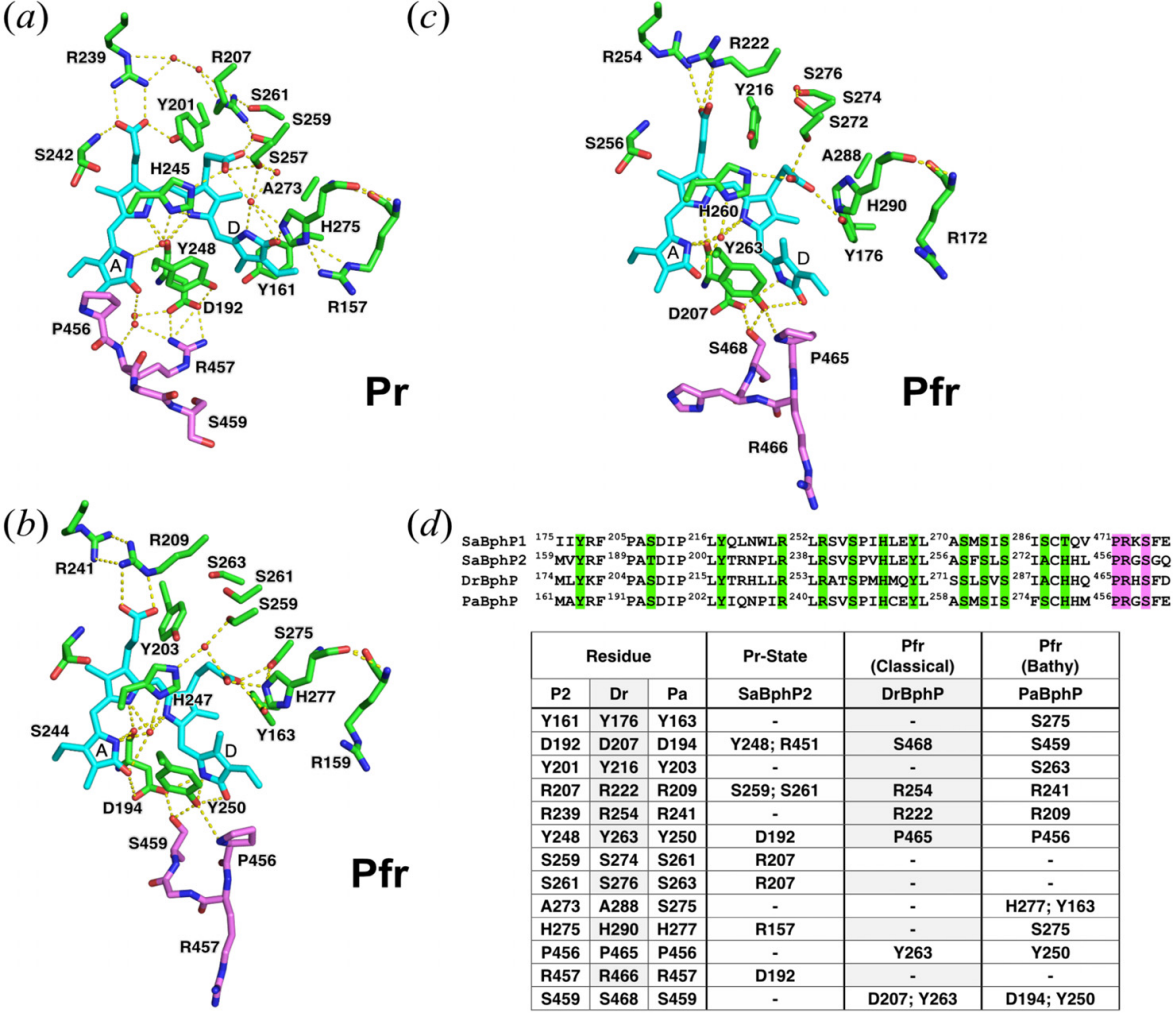
PRXSF motif of the PHY domain and its specific hydrogen-bonds with the amino acids of the GAF domain in the Pr state of *Sa*BphP2 wild-type (a) and Pfr states of bathy *Pa*BphP (b) and classical *Dr*BphP (c) phytochrome proteins. (d) Partial protein sequence alignment highlighting critical amino acids in the GAF (green) and PHY (magenta) domains that form hydrogen bond interactions (highlighted with dashed lines). The table summarizes amino acid interactions of GAF and PHY domains of *Sa*BphP2 (Pr), classical *Dr*BphP (Pfr), and bathy *Pa*BphP (Pfr).

The PHY domain harbors the sensory tongue [Fig. 1, amino acids 435–485, Figs. 3(a)–3(c)] that is in direct contact with the GAF domain. The sensory tongue reacts to the rotation of ring D caused by the isomerization of the C15=C16 double bond. It is transducing the light induced signal to the rest of the PHY and the output domains.^8,21–23,43–47^ Although the helical kink is absent in all three *Sa*BphP2 structures reported here, the position of the sensory tongue remains essentially identical in wild-type PCM of *Sa*BphP1 and *Sa*BphP2.

The cryostructure of the His275Thr mutant is essentially identical to that of the wild-type (supplementary material Table 2), except at the location of the point mutation (supplementary material Fig. 4) where the hydrogen bond to the ring D carbonyl is absent. Given the high resolution of *Sa*BphP2 wild-type structure, we clearly observe an intricate network of waters and conserved amino acids which stabilize the BV-chromophore in the Pr state. This network includes residues from including the PASDIP motif in the GAF domain and the PRXSF motif of the PHY domain [Figs. 2(c) and 3(d)]. Interestingly, not a single amino acid from the PHY domain forms direct contact with the BV chromophore. This is somewhat surprising knowing that the PHY domain has been reported as essential for formation of the Pfr state.^20,48^ Constructs lacking the PHY domain, known as chromophore-binding domains (CBDs), are partially photoactive. They show a much smaller shoulder in the Pfr form spectrum similar to constructs that lack the conserved Histidine which would (if present) stabilize the D-ring in the Pr state [Fig. 2(a) and supplementary material Fig. 2]. Similar photoactivity was observed for the *Sa*BphP2 His275Thr mutant and the *Sa*BphP1 wild-type protein (Thr289 instead), both lacking the conserved Histidine [Fig. 2(b)].^5,14,18,48,49^

We note that the PRXSF motif of the PHY domain undergoes significant structural changes from Pr to Pfr. The proline in this motif shifts from a position near ring A to being closer to the D-ring. This Pro specifically forms an important network of hydrogen bonds with the Y263 and the D-ring carbonyl upon isomerization of the C15=C16 double bond and formation of the stable Pfr form [Figs. 3(b) and 3(c)]. Interestingly, the only phytochrome that forms a Pnr (near-red) state upon red-light illumination, *R. palustris Rp*BphP3, lacks the conserved Proline and has Threonine instead. A single Thr475Pro mutant in *Rp*BphP3 generates a classical Pr/Pfr BphP that reversibly switches between Pr and Pfr states after 660 and 750 nm illumination, respectively.^8,20^

## DISCUSSION

### Dimer interface and its role in signaling

**FIG. 4.**
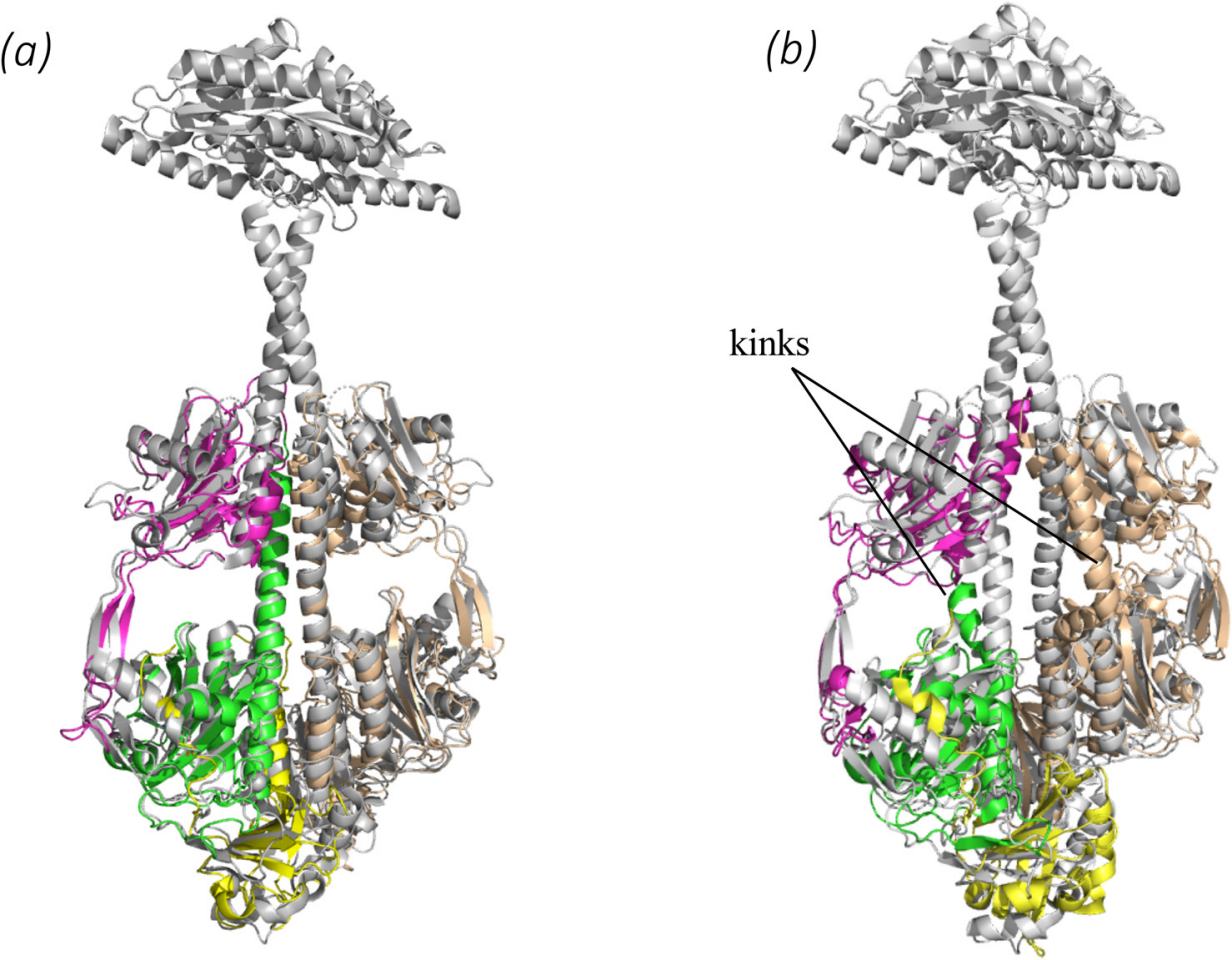
Structural comparison with important BphPs. (a) Superposition of the *Sa*BphP2 PCM (Pr) (yellow, green, and magenta) onto the full length BphP with a diguanylyl cyclase effector domain from *Idiomarina* sp. (gray).^46^ (b) Superposition of the *Sa*BphP1 PCM (yellow, green, and magenta) on the full length BphP with a diguanylyl cyclase effector domain from *Idiomarina* sp. (gray).^46^ The helical kink is absent in (a) and marked in (b). In both *Sa*BphPs, the sensory tongue displays a β-sheet structure indicative of the Pr state.

Crystal structures of the wild-type classical *Sa*BphP2 determined at the highest resolution of any PCM to date reveal an intricate network of hydrogen bonds between conserved amino acids, neighboring water molecules, and BV which all stabilize the Pr state. To our surprise, the dimer interface structure of *Sa*BphP2 is different from those of the related classical phytochromes such as *Dr*BphP^21,50^ and *Rp*BphP2.^8^ They all have an extensive helical kink at the dimer interface that is absent from *Sa*BphP2. We superimposed the *Sa*BphP2 structure onto the only x-ray structure of an intact BphP containing diguanylyl cyclase enzymatic domain [Fig. 4(a)]. Interestingly, *Sa*BphP2 fit nearly perfectly onto the PCM of the *Idiomarina* BphP.^46^ Comparing the structures of *Sa*BphP1 and *Sa*BphP2 in Fig. 1, it is suggestive that if the kink is straightened out in P1, its structure would be essentially identical to the P2 structure. A similar kink is also observed in other BphP structures such as the *Dr*BphP^50,51^ and to a smaller extent in *Rp*BphP2^8^ and is likely caused by crystal contacts and lattice constraints. By comparison with the *Idiomarina* full length structure (Fig. 4), a more biologically relevant PCM structure would be the one with straight helices as shown here for the *Sa*BphP2.

### Origin and propagation of the light signal

**FIG. 5.**
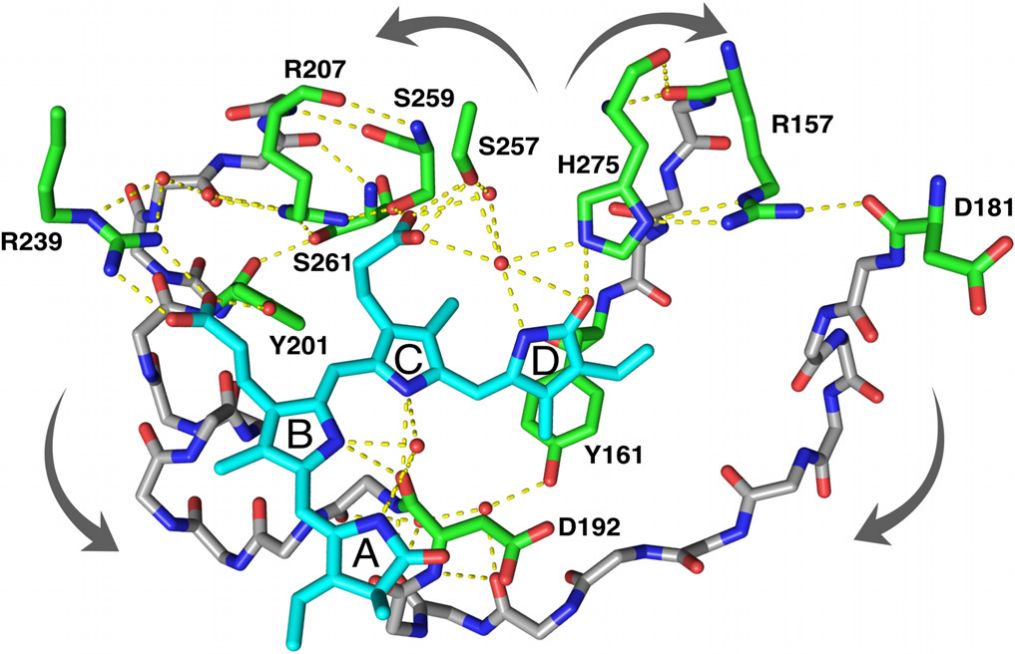
Two proposed pathways for signal transduction highlighted in the Pr state of *Sa*BphP2 wild-type protein. His 275 together with the conserved Arg 207 and Arg 157 undergoes significant conformational changes upon Pfr formation as observed in the classical *Dr*BphP and the bathy *Pa*BphP [Figs. 3(b) and 3(c)]. The protein backbone connecting to the conserved PASDIP motif and D192 is shown in gray.

Upon Z to E isomerization and by rotation of the D-ring about the C15=C16 double bond, the conserved Histidine 275 must move. This destabilizes interactions with the neighboring amino acids and the water network in two ways, each involving a conserved Arg (Arg 157 and Arg 207) and a conserved Tyr (Tyr 161 and Tyr 201) as gateways of the signal (Fig. 5). One pathway is via the conserved R157 and consequently the main protein backbone. This involves the conserved Y161 preceding the important PASDIP motif and results in significant movement of D192. The second pathway is via the water network and R207. This disrupts the hydrogen bond with the conserved Y201, which stabilizes the B-ring propionate and ultimately changes the structure of the main protein backbone beyond the PASDIP motif. As a consequence, the salt bridge between Aspartate 192 in PASDIP and Arginine 457 from the PRXSF motif of the C-terminal PHY domain should be affected. Not surprisingly, this Arginine 457 is completely removed from the BV-binding pocket area in the Pfr state as observed in the classical *Dr*BphP and bathy *Pa*BphP. Instead of Arg 457, in the Pfr state, the Serine 459 from the same PRXSF motif forms a hydrogen bond with Asp 192 due to the newly formed helix in the sensory tongue region.

It is important to highlight that only a few amino acids after the PRXSF motif a short (C-terminal) helix is present that extends into a long helical backbone in intact (full length) phytochromes. This linker connects to the effector, enzymatic domain as seen in the intact structure of *Idiomarina* BphP. Gourinchas *et al.*^45,46^ introduced specific mutations in the coil-coil linker interface of *Idiomarina* BphP. This results in a heterogeneous BphP with one monomer in the Pr and other monomer in the Pfr state which is enzymatically active (but lost light regulation). This further supports that the dimer interface plays an essential role in the generation of the signal and the regulation of the enzymatic activity.

### Histidine 275 and D-ring rotation

The conserved Histidine, although absent in some myxobacterial BphPs such as *Sa*BphP1, stabilizes the Pr state by forming a H-bond with the D-ring carbonyl and the Pfr state by forming a H-bond with the C-ring propionate. Previously it has been suggested that protonation of His 275 and deprotonation of the C-ring propionate are critical in determining the reactivity of the BV-chromophore.^49,52^ Upon protonation, His 275 supports the movement of the C-ring propionate toward ring D, which leads to a rearrangement of the hydrogen bond network. However, there is an important Serine [Ser 287 in *Sa*BphP1 or Ser 275 in *Pa*BphP, Fig. 3(b)] that is in proximity to the conserved Histidine and that is found in some BphPs, particularly in those that lack Histidine and contain Threonine (Thr 289 in *Sa*BphP1) or Glycine instead.^5,49^ In the bathy *Pa*BphP, both Serine and Histidine stabilize the C-ring propionate through a hydrogen bond in the Pfr state,^23^ while in the *Sa*BphP1 His289Thr mutant, both Serine and Histidine form a hydrogen bond to the D-ring carbonyl in the Pr state [Figs. 2(a), 2(b), and 3(a)–3(c)].^5,14,18^ In the *Sa*BphP1 wild-type that lacks this conserved Histidine, Serine is 3.6 Å away from carbonyl of the D-ring with which it likely forms a H-bond. This is supported by the Pr/Pfr photoactivity of the *Sa*BphP1 wild-type.^5,14^ Serine is absent in the *Sa*BphP2 wild-type protein, and likewise, the mutation His275Thr diminishes the Pr/Pfr photoactivity (supplementary material Fig. 2), possibly as there is no stabilizing amino acid for the D-ring and C-ring propionate in the Pr and Pfr states.

### *Sa*BphP2 as a model for time-resolved crystallographic experiments

**FIG. 6.**
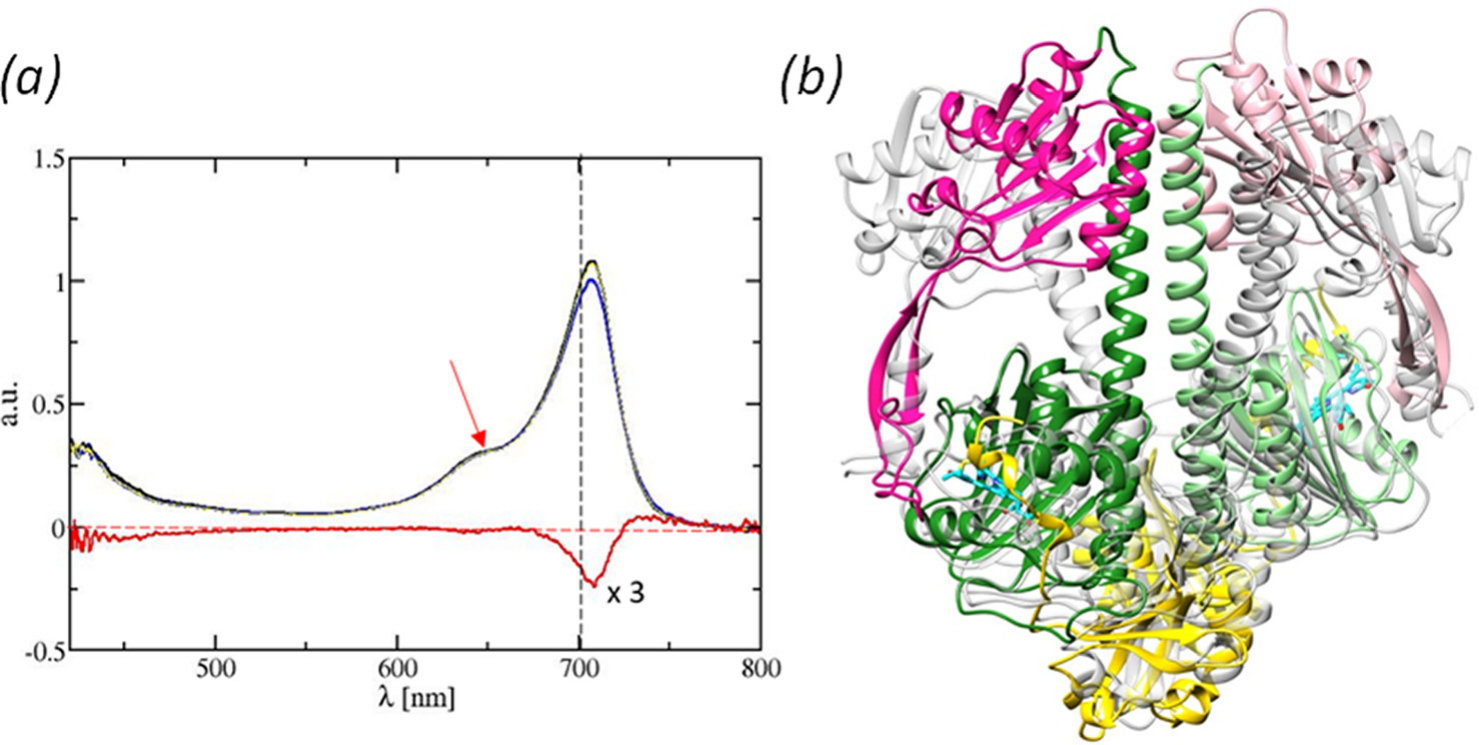
Photoactivity of *Sa*BphP2 crystals and expected protein structural changes upon red-light illumination. (a) Absorption spectra of the crystals of wild-type *Sa*BphP2 PAS-GAF-PHY before and after light illumination. Black line: spectrum in the dark; blue line: spectrum after 1 min of illumination with a 660 nm light emitting diode at ∼5 mW/mm^2^. Spectral changes are reversible. Red line: difference spectrum (3× enhanced). Dashed red line: zero line. Red arrow: illumination wavelength. (b) Superposition of the *Sa*BphP2 PCM (Pr) (yellow, green, and magenta) onto the *Dr*BphP PCM (Pfr) in gray.^21,22^ The PHY domains are displaced substantially in the Pfr structure.

An ambitious goal is to observe the *Z* to *E* isomerization of the phytochromes by time-resolved crystallographic methods and characterize the transmission of signal from the BV to the enzymatic domain, ultimately through a distance of about 150 Å of protein, in real time. The *Sa*BphP2 PCM is photoactive and displays a stable Pfr state that is long(er) lived in solution and also in the crystal [Fig. 6(a)]. Structural changes of the entire protein backbone from Pr to Pfr may be extensive [Fig. 6(b)]. Our spectra suggest that time-resolved serial femtosecond crystallography experiments are feasible with various pump-probe time delays with *Sa*BphP2 crystals spanning from the ultrafast to slower time scales. In this study, the *Sa*BphP2 wild-type microcrystals diffracted to 2.1 Å at SACLA in nuclear grade grease [supplementary material Fig. 1(b)]. At this resolution, difference electron-density maps most likely contain chemically meaningful features even at low population transfer rates^53–56^ and can be used to identify and characterize the structures of at least some of the transient intermediates during the Pr to Pfr transition.

## SUPPLEMENTARY MATERIAL

See the supplementary material for two tables and four figures. Supplementary material Table 1 lists data collection and data refinement statistics and supplementary material Table 2 lists distances between selected atoms in the reported structures. Supplementary material Fig. 1 shows micro and macrocrystals of *Sa*BphP2, supplementary material Fig. 2 reports absorbance spectra of wild-type and His275Thr mutant of *Sa*BphP2, supplementary material Fig. 3 compares the chromophore binding pocket of the three *Sa*BphP2 structures, and supplementary material Fig. 4 shows electron density maps for various regions in the reported structures.
